# Nonlinear dynamics of early atherosclerotic plaque formation may determine the efficacy of high density lipoproteins (HDL) in plaque regression

**DOI:** 10.1371/journal.pone.0187674

**Published:** 2017-11-21

**Authors:** Alexander D. Chalmers, Christina A. Bursill, Mary R. Myerscough

**Affiliations:** 1 School of Mathematics and Statistics, The University of Sydney, Sydney, NSW, Australia; 2 South Australian Health and Medical Research Institute, Adelaide, South Australia, Australia; Universiteit Gent, BELGIUM

## Abstract

We use a computational model to explore the effect of foam cell accumulation on plaque regression following an increase in high density lipoprotein (HDL) influx into the plaque. Atherosclerotic plaque formation is the outcome of cellular and cytokine responses to low density lipoproteins (LDL) that penetrate the artery wall following an injury to the endothelium and become modified. We modelled the cells and cytokines that are most important in plaque formation using partial differential equations. The model includes monocytes and macrophages, foam cells, macrophage chemoattractants, endothelium-stimulating cytokines, modified low density lipoproteins (mod LDL) and HDL. We included interactions both at the endothelium surface and inside the artery wall. The model predicts that when HDL influx into a well-established plaque with large numbers of foam cells is increased, the plaque may not regress but may continue to grow at a slower rate. If HDL influx is increased when a model plaque is recently established and has fewer foam cells, then the plaque does regress. If modLDL influx into the plaque is lowered at the same time that HDL influx increased or the capacity of the HDL to remove cholesterol from foam cells is increased, then the plaque is more likely to regress. The predictions of the model are in qualitative agreement with experimental studies in mice and rabbits. The results suggest that the intrinsic dynamics of reverse cholesterol transport by HDL are important in determining the success of HDL raising in promoting plaque regression.

## Introduction

There is a well-known correlation, at a population level, between high levels of high density lipoprotein cholesterol (HDL-C, often called “good cholesterol”) in the blood plasma and a reduced risk of cardiovascular disease in humans [1–3]. Following disappointing results from large clinical trials of drugs that are known to raise blood HDL-C [4–7], it has become clear that it is the number of HDL particles [8, 9] and their efficacy [9, 10] that correlate most closely with the risk of cardiovascular disease. Levels of HDL-C, the cholesterol carried by HDL particles, broadly correlate with the number of HDL particles [9] but raising the amount of cholesterol carried by HDL particles does not necessarily result in an increase in the total number of HDL particles and so may not result in increased action by HDL [11, 12]. In this paper we explicitly focus on HDL particles which act to remove lipid from macrophages and not on HDL cholesterol (HDL-C).

There is experimental evidence from animal models which suggests that raising HDL may lead to plaque regression in recently established plaques when the number of HDL particles is increased, [13–15], but not in older plaques [16–19]; it will only reduce the rate of progression in these older plaques.

As plaques grow, they may develop lipid cores, calcification and collagen caps which early plaques do not have. These structures still allow cholesterol to be removed from the plaque, leading to regression in late stage plaque [16, 18] which suggests that structures typical of late stage plaques do not prevent lipid from entering or exiting plaques. It is likely, therefore, that the failure of increased HDL in the bloodstream to promote plaque regression in late stage plaque is due to something other than the plaque becoming impermeable to lipoproteins due to changes in its structure.

Plaque formation and growth are complicated and include many interactions between cells, lipids, cytokines and other factors [20]. Many of these interactions are nonlinear; that is, the reaction rate is not proportional to the concentration or density of the inputs. For example, the uptake rate of modLDL by macrophages will saturate as the concentration of modLDL increases, because the rate that each macrophage can internalise modLDL is inherently limited by the number of receptors in the cell membrane or even by the area of the cell membrane itself. Therefore as modLDL availability in the artery wall increases, the rate that modLDL is removed by macrophages will not necessarily remain proportional to modLDL availability.

The consequences of nonlinear interactions are well known in biological systems such as ecology and neuroscience [21, 22]. The state of the system may undergo sudden dramatic switches in response to a small change in inputs or the effect of a change in inputs may depend on the current state of the system—for example, a change may produce an effect early in the development of the system, but not later. Mathematical and computational modelling of such systems shows that these effects are not random but qualitatively predictable.

We propose a computational model for plaque growth which includes the action of HDL and predicts that plaques will not regress if HDL particle influx into the plaque is increased too late in plaque development. In our model we consider only plaques which do not have necrotic cores, but may contain large numbers of foam cells and macrophages. We define regression in this model as a reduction in the number of inflammatory macrophages and foam cells. The failure of HDL to promote plaque regression in the model is due purely to the inherent dynamics of plaque growth and of HDL action. Increasing functional HDL influx too late in plaque development only attenuates plaque growth and does not enable plaque regression.

Reducing the size of necrotic cores in advanced plaques is a major goal of clinical intervention and therapy, particularly following an ischaemic event such as a heart attack or stroke. The model presented here only addresses the reduction in foam cell numbers and therefore decreasing the amount of intracellular lipid in the plaque, rather than the removal of the extracellular lipid found in necrotic cores. Free lipid that is removed from necrotic cores by macrophage phagocytosis, however, will become intracellular lipid as an intermediate step in its removal from the plaque. In this way, macrophages which contain lipid ingested from the necrotic core may also be part of the foam cell population. This model, therefore, may give some indirect insight into necrotic core regression by addressing the removal of ingested lipid from foam cells by HDL.

Previous mathematical and computational models for plaque development that include the action of HDL have focused on the relative importance of different functions of HDL [23] or predicting risk as a function of the balance between HDL-C and LDL-C [24]. Here we focus specifically on the impact of the timing of changes in the rate of HDL influx into the plaque on plaque development and regression.

## Modelling and methods

The model that we present here focuses on immunological processes within the artery wall. Many of the immunological processes included in the model are inherently *in vivo* processes and difficult to measure directly. This model, therefore, gives qualitative insight only because of the difficulty in obtaining data to fit the parameters required for a fully quantitative model. Nevertheless qualitative models provide valuable insights, generate hypotheses and motivate the data collection that is needed to produce fully quantitative models.

We emphasise that this model is not concerned with fluid flow within the blood vessel [25] nor with the details of the relationship of LDL and macrophage perfusion to endothelial dysfunction [26]. Both of these aspects are well-studied with models and experimentally. Instead, we assume that the endothelium (the layer of cells that line the inside of the blood vessel) is sufficiently damaged to permit plaque formation and we model only the immunological processes inside the artery wall.

**Fig 1:**
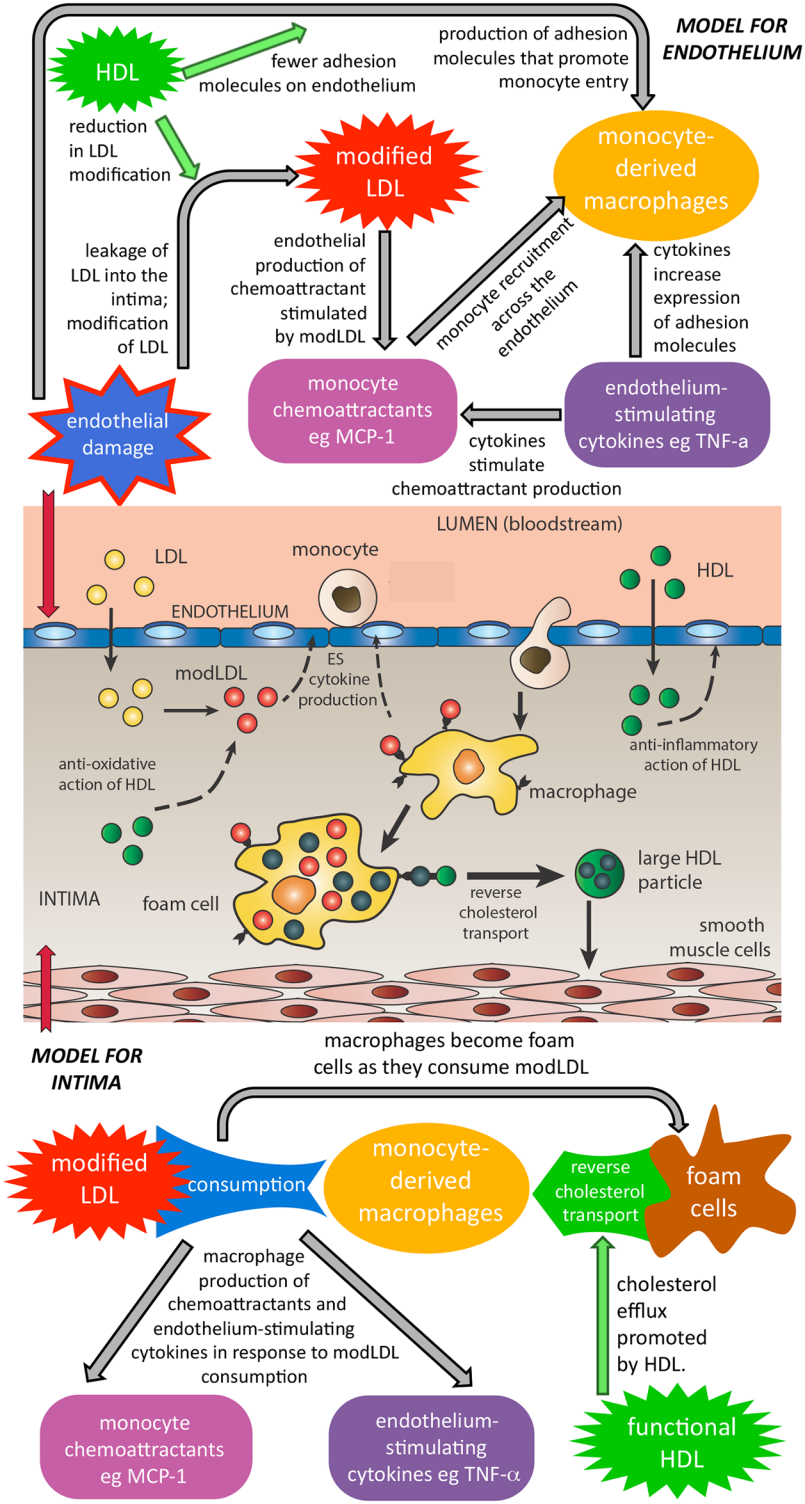
Schematic diagram of the model. Diagram of processes in the endothelium and intima in early plaque formation with a flow-chart representation of the model interactions on the endothelium (top) and in the intima (bottom) between modLDL, monocytes/macrophages, chemoattactants, ES cytokines, foam cells and HDL. A plaque is initiated when the endothelium is injured and allows LDL to enter and causes it to become modified so that the LDL particles become oxidised or modified in other ways. These modLDL particles provoke an immune reaction that causes monocytes to enter the blood vessel wall from the blood stream and differentiate into macrophages which consume modLDL. The macrophages become filled with cholesterol and take on a foamy appearance under the microscope. If the cholesterol is not removed from the cells, these foam cells accumulate in the intima. HDL particles transport cholesterol out of foam cells and cause the plaque either to regress or grow more slowly. HDL also acts to reduce the modification of LDL and the excitation of the endothelium which reduces the rate that monocytes enter the plaque.

Fig 1 shows schematically how the model works. We assume that the injured endothelial cells allow LDL into the intima, the layer of the artery wall nearest to the bloodstream. This LDL rapidly becomes modLDL and stimulates an immune reaction in the endothelial cells on the boundary. In response to modLDL, endothelial cells produce monocyte chemoattractants such as MCP-1, which draw monocytes into the intima. Endothelium-stimulating (ES) cytokines such as TNF-*α*, produced in the intima, stimulate the endothelial cells to produce adhesion molecules which act on monocytes in the blood stream and further increase the rate of monocyte recruitment. Inside the intima the monocytes rapidly differentiate into macrophages which move towards modLDL, and consume it. Stimulated by modLDL consumption, macrophages produce more monocyte chemoattractant and also ES cytokines. As macrophages consume modLDL, they become foam cells. The model applies to plaque development before the formation of the lipid core or collagen cap.

HDL modulates these processes in the model by inhibiting the oxidation of LDL so that there is less modLDL entering the intima; by decreasing the number of adhesion molecules on the damaged endothelium, which in turn reduces the recruitment rate of monocytes; and by facilitating reverse cholesterol transport (RCT) from foam cells which then revert to macrophages. Macrophages have the capacity to change phenotype during plaque progression and regression [27, 28]. There is provision in the model fora proportion of the macrophages to revert to the original inflammatory phenotype (M1) following RCT. When macrophages revert to the anti-inflammatory (M2) phenotype, they no longer play any part in the model as we assume that they no longer produce pro-inflammatory cytokines such as MCP-1 and ES cytokines. HDL can also promote foam cell and macrophage emigration from the plaque [29]. This emigration from the plaque can be modelled in the same way as loss of macrophages from the model through change of phenotype. We assume that some proportion leave the plaque after lipid has been removed by HDL and those macrophages whose fate is emigration are no longer part of the model.

The model consists of six partial differential equations with associated boundary conditions. We solved the equations in one spatial dimension which is taken as a radial line from the endothelium to the boundary between the intima and the media which is the next outward layer of the artery wall. This assumes that the model domain is far enough away from the edges of the plaque that there is negligible net movement of cells and cytokines either circumferentially or axially in the plaque. The endothelial boundary, between the artery wall and the bloodstream is set at *x* = 0. The spatial dimension is scaled by the width of the intima, so that the medial boundary is at *x* = 1. To keep the computations tractable, we assumed that this domain is fixed in length and so the plaque does not significantly distort the intima. This is a reasonable approximation for early plaque but may not be a good approximation for later plaques.

The six dependent variables are *ℓ*, *h*, *p* and *q*, the concentrations of modLDL, HDL particles with capacity for RCT, monocyte chemoattractant and ES cytokine respectively and *m* and *N*, respectively the density of macrophages/monocytes and foam cells in the tissue. We assume that the timescale of LDL modification is much faster than the timescales of other events in the model [30] so that we only model the LDL which will become modified on entry to the intima and we ignore the LDL that enters the intima but remains unmodified. We also model macrophages and the monocytes that will differentiate into macrophages that ingest modLDL as a single class of cells in the model.

The model has a large number of parameters. The definition and symbol for each of these parameter are given in Table 1 together with the numerical value used in numerical computation.

**Table 1:** Parameter values used in the model. It is difficult to obtain experimentally valid parameter values for all the parameters in the model and consequently these values are not available in the literature. In particular it is extremely hard to measure *in vivo* values, but most of the parameter that the model requires have not even been measured *in vitro*. Therefore, most values are order of magnitude estimates. Since the aim of this study is to produce qualitative results, having exact, experimentally determined parameter values is less critical than for a quantitative predictive model. The values below have been rescaled in space and time in order to normalise the intima width. We take an intima width of 40 *μ*m [59]. We use a time scale of ∼ 7.7 × 10^6^ s (approximately 89 days) to rescale the equations. Further details on the rescaling can be found in [58].

Parameter	Value after rescaling	Reasoning/Description
*D*_*ℓ*_	10^4^	Diffusion of LDL paricles ∼ 2 *μ*m^2^/s. [60]
*μ*_*ℓ*_	10^5^	Estimated consumption rate of LDL by macrophages.
*d*_*ℓ*_	10^1^	Estimated decay rate of LDL.
*σ*_*ℓ*_	10^3^	Estimated influx rate of LDL.
*D*_*p*_	10^6^	Diffusion of chemoattractant ∼ 200 *μ*m^2^/s. [61]
*μ*_*p*_	10^6^	Estimated production rate of chemoattractants by macrophages consuming LDL.
*d*_*p*_	10^3^	Decay rate of chemoattractant ∼ 10^−4^/s. [62]
*σ*_*p*_1__	10^5^	Estimated production rate of chemoattractants by endothelial cells by LDL stimulation.
*σ*_*p*_2__	10^4^	Estimated production rate of chemoattractants by endothelial cells by ES cytokine stimulation.
*β*_*p*_	10^0^	Estimated saturation constant of LDL stimulation of endothelial cells in the production of chemoattractant.
*D*_*m*_	10^2^	≪ *D*_*ℓ*_. Estimated random movement of macrophages.
*χ*_*m*_	10^3^	Estimated chemotactic term due to scavenging of LDL.
*μ*_*m*_	10^2^	≪ *μ*_*ℓ*_. Estimated coversion rate of macrophages to foam cells via LDL consumption.
*d*_*m*_	10^0^	Estimated decay rate of macrophages.
*σ*_*m*_	10^−3^	Estimated adhesion efficacy on inward migration of monocytes into the intima.
*A*	10^−2^	Estimated efficacy of ES cytokines on the transmigration of monocytes into the intima.
*P*_0_	10^−1^	Estimated background chemoattractant levels.
*D*_*q*_	10^6^	Diffusion of chemoattractant ∼ 200 *μ*m^2^/s. [61]
*μ*_*q*_	10^6^	Estimated production rate of ES cytokines by macrophages consuming LDL.
*d*_*q*_	10^3^	Decay rate of chemoattractant ∼ 10^−4^/s. [62]
*σ*_*q*_	10^0^	Estimated outflux of ES cytokines
*D*_*N*_	10^−2^	≪ *D*_*m*_. Estimated random movement of macrophages.
*ν*_*N*_	10^1^	Estimated coversion rate of foam cells to macrophages via HDL reverse cholesterol transport.
*D*_*h*_	10^4^	Assumed same as *D*_*ℓ*_.
*ν*_*h*_	10^4^	Estimated consumption rate of HDL through reverse cholesterol transport.
*d*_*h*_	10^1^	Estimated decay rate of HDL.
*κ*	10^0^	Estimated saturated constant of HDL in reverse cholesterol transport.
*σ*_*h*_	Varies	Estimated influx of HDL into the intima.
*α*_*ℓ*_	10^1^	With *γ*_*ℓ*_, estimated efficacy of HDL on the oxidation of LDL.
*γ*_*ℓ*_	10^−1^	≪ *α*_*ℓ*_.
*α*_*m*_	10^1^	With *γ*_*ℓ*_, estimated efficacy of HDL on the oxidation of LDL.
*γ*_*m*_	10^−1^	≪ *α*_*m*_.

We assume that each macrophage consumes modLDL at a rate that is proportional to the concentration of modLDL when modLDL concentration is low. When modLDL concentration is high, the rate of consumption tends to a fixed upper limit [31]. In the model, these saturating kinetics for modLDL consumption by a single cell are represented by the function *ℓ*/(1 + *ℓ*) where *ℓ* has been scaled so that the constant in the denominator is 1. This uptake rate, which is essentially Michaelis-Menten kinetics, is the simplest possible saturating function.

The first term on the right hand side in each of Eqs (1) to (6) models the diffusion or random motion of the lipid particles, cytokines or cells in the tissue. We acknowledge that many of these species may undergo binding to other substrates within the tissue which constrains their ongoing movement. However Fickian diffusion is a reasonable representation of movement before binding takes place [32] and has the added mathematical benefit of fostering stable numerical solutions to the model equations.

The last term on the right hand sideof each of Eqs (1) to (5) models linear loss due to chemical decay, cell apoptosis or other processes not explicitly included in the model.

In Eq (1) for the rate of change of modLDL, concentration *ℓ*, the second term on the right hand side models the loss of modLDL due to consumption by macrophages:

$$\begin{array}{c} \frac{\partial \ell }{\partial t}=D_{\ell }\frac{\partial^{2}\ell }{\partial x^{2}}-\mu_{\ell }\frac{\ell m}{1+\ell }-d_{\ell }\ell \;. \end{array}$$ (1)

The second term on the right hand side of Eq (2) for the change in concentration of HDL, *h*, models loss of those HDL particles which have the capacity to take up lipid as HDL particles receive lipids via RCT from foam cells. This term saturates as *h* increases, since each foam cell has a finite number of transporters in its membrane and so there is a limit to the rate that each cell can engage with HDL particles. The equation for the rate of change of the concentration of HDL is, therefore:

$$\begin{array}{c} \frac{\partial h}{\partial t}=D_{h}\frac{\partial^{2}h}{\partial x^{2}}-\nu_{h}\frac{hN}{\kappa +h}-d_{h}h\;. \end{array}$$ (2)

For chemoattractant, concentration *p*, and ES cytokine, concentration *q*, the second term on the right hand sides of Eqs (3) and (4) models production of these cytokines by macrophages in response to macrophage consumption of modLDL [33, 34]. This rate of production in the model is, therefore, proportional to the rate that macrophages consume modLDL:

$$\begin{array}{c} \frac{\partial p}{\partial t}=D_{p}\frac{\partial^{2}p}{\partial x^{2}}+\mu_{p}\frac{\ell m}{1+\ell }-d_{p}p\;; \end{array}$$ (3)

$$\begin{array}{c} \frac{\partial q}{\partial t}=D_{q}\frac{\partial^{2}q}{\partial x^{2}}+\mu_{q}\frac{\ell m}{1+\ell }-d_{q}q\;. \end{array}$$ (4)

Macrophages, density *m*, move towards modLDL (second term in Eq (5)) [33] and convert to foam cells as they consume modLDL (third term). Only a proportion *θ* of foam cells revert to macrophages with an inflammatory phenotype after RCT (fourth term). Some macrophages take on a non-inflammatory phenotype after exposure to HDL [35] and we do not include this macrophage phenotype in the model. Other macrophages leave the plaque after RCT [29] and are also not included in the model once their fate is determined so only a proportion *θ* of foam cells that undergo RCT return to the pool of inflammatory macrophages. Hence the equation for macrophages, assuming that all have an inflammatory phenotype is:

$$\begin{array}{c} \frac{\partial m}{\partial t}=D_{m}\frac{\partial^{2}m}{\partial x^{2}}-\chi_{m}\frac{\partial }{\partial x}\;(m\frac{\partial \ell }{\partial x})-\mu_{m}\frac{\ell m}{1+\ell }+\theta \nu_{N}\frac{hN}{\kappa +h}-d_{m}m\;. \end{array}$$ (5)

Foam cells, density *N*, are generated as macrophages consume modLDL (second term, Eq (6)) and revert to macrophages as HDL removes lipids (third term):

$$\begin{array}{c} \frac{\partial N}{\partial t}=D_{N}\frac{\partial^{2}N}{\partial x^{2}}+\mu_{m}\frac{\ell m}{1+\ell }-\nu_{N}\frac{hN}{\kappa +h}\;. \end{array}$$ (6)

At the endothelial boundary, *x* = 0, the boundary conditions model the activity of the endothelium. HDL flows into the plaque, ES cytokines flow outwards at a rate proportional to the concentration of ES cytokines in the intima, foam cells do not cross this boundary:

$$\begin{array}{c} J_{h}=\sigma_{h}\;; \end{array}$$ (7)

$$\begin{array}{c} J_{q}=-\sigma_{q}q\;; \end{array}$$ (8)

$$\begin{array}{c} J_{N}=0\;. \end{array}$$ (9)

where *J*_*h*_, *J*_*q*_ and *J*_*N*_ are the inward flux of HDL, ES cytokines and foam cells respectively across the boundary. The flux of modLDL across *x* = 0 into the intima, *J*_*ℓ*_, is modulated by the presence of HDL which reduces LDL oxidation [36]:

$$\begin{array}{c} J_{\ell }=\sigma_{\ell }\frac{1+h/\alpha_{\ell }}{1+h/\gamma_{\ell }}\;. \end{array}$$ (10)

The flux of monocyte chemoattractant *J*_*p*_ into the intima is determined by the rate that it is produced by endothelial cells. This production is a function of ES cytokine concentration [37] and modLDL concentrations [38]:

$$\begin{array}{c} J_{p}=\sigma_{p_{1}}\frac{\ell }{\beta_{p}+\ell }+\sigma_{p_{2}}q\;. \end{array}$$ (11)

The inward flux of macrophages is dependent on endothelial excitation by ES cytokines, by the action of monocyte chemoattractants and the anti-inflammatory effects of HDL [36, 38]:

$$\begin{array}{c} J_{m}=\sigma_{m}\frac{1+h/\alpha_{m}}{1+h/\gamma_{m}}\left(1+Aq\right)\left(p-P_{0}\right)H\left(p-P_{0}\right), \end{array}$$ (12)

where *H*(*x*) is the Heaviside function and *P*_0_ is the concentration of monocyte chemoattractant in the lumen of the artery.

All variables have no flux boundary conditions on *x* = 1; that is, we assume that there is no significant loss of cells or cytokines through the media. This does not prevent lipid-laden HDL passing out through the outer wall of the blood vessel as once an HDL particle loses the ability to accept lipid it is no longer included in the model—this is expressed in the loss term which is the last term on the right hand side of Eq (2)—and so the no flux assumption does not apply to lipid-laden HDL. In the same way, macrophages that emigrate from the plaque or change to a non-inflammatory phenotype cease to be part of the model and so do not require a boundary flux term in the model. We are able to model macrophage emigration in this way since we do not track the volume of cell or lipids in this model.

The set of partial differential equations (PDEs) Eqs (1–6) with boundary conditions (7–12) were solved for time-dependent solutions using the software package FlexPDE which uses finite element methods to solve systems of PDEs. The bifurcation curves for the steady states of the set of equations were found numerically using the numerical continuation package AUTO [39]. At the steady state, this system of PDEs reduces to an equivalent system of ordinary differential equations (ODEs) in the independent spatial variable *x*. We first used FlexPDE to find the values for each dependent variable across the discretised spatial domain, for a value of *σ*_*h*_ that guarantees a steady state for the foam cell population. Using these values as a starting point we applied AUTO to the ODE system, to find the bifurcation curve as a function of *σ*_*h*_. The complete set of parameter values used in these computations are given in Table 1.

It is possible to formulate models for plaque development which have a moving boundary that enables the increasing numbers of foam cells to distort the intima [40]. Here we are primarily concerned with HDL dynamics rather than intimal thickening and we have made the simplifying assumption that the domain is fixed; that is, that the influx of modLDL and macrophages do not significantly distort the intima.

## Results

**Fig 2:**
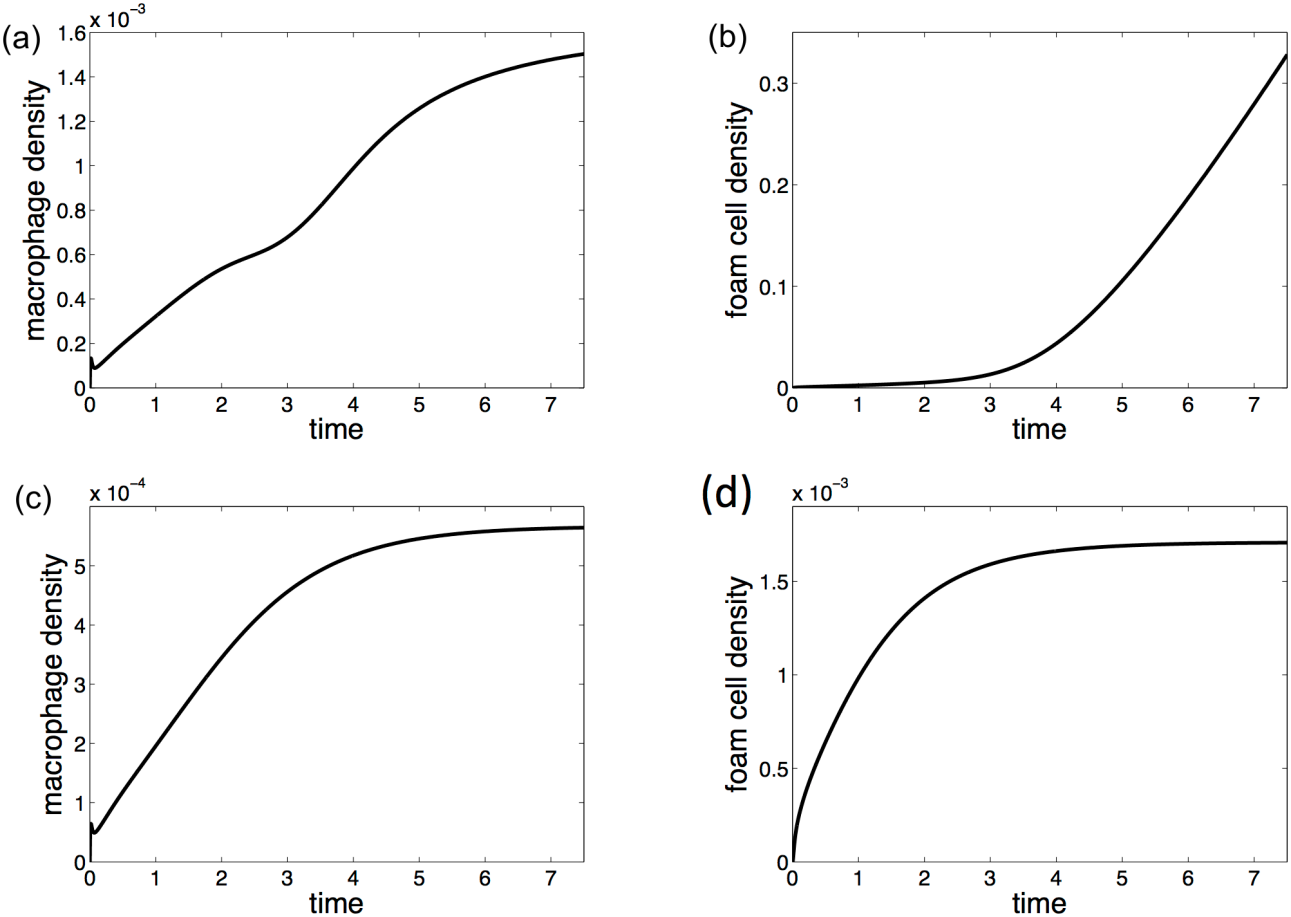
Macrophage and foam cell density over time. (a) Macrophage density in the intima and (b) foam cell density when the plaque grows unboundedly. Plots (c) and (d) show macrophage and foam cell density respectively where foam cell numbers settle to a fixed equilibrium. Note: all variables are scaled with respect to intima width and indicative time scale.

The solutions of the model produce spatial profiles across the intima of modLDL concentration, and macrophage and foam cell density. These profiles change over time as the plaque develops. The model plaques start with no modLDL, cytokines, inflammatory macrophages or foam cells present in the intima. At first, each profile has a maximum at, or close to, the endothelium. As the plaque develops, both cells and cytokines become evenly spatially distributed across the intima. The density of macrophages in the intima in these model plaques always settles to a fixed equilibrium, which does not change after an initial period of growth, but the density of foam cells may either continue to grow or settle to a fixed equilibrium where the plaque is neither growing nor shrinking (Fig 2).

**Fig 3:**
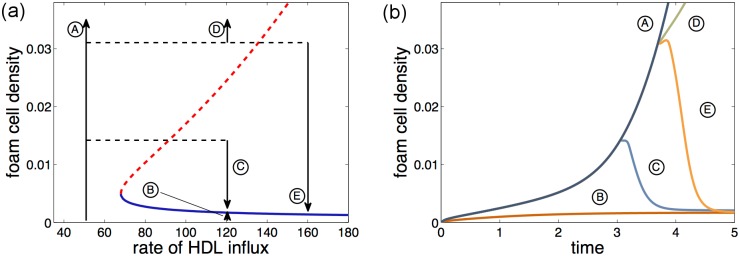
Model predictions from bifurcation diagram. (a) Bifurcation diagram showing the density of foam cells at equilibrium as a function of the rate of influx of HDL. The solid blue curve represents the attracting equilibrium and the dashed red curve the repellor. The black arrows and lines represent various plaques labeled *A*–*E* whose size changes with time; the dashed horizontal lines represent rapid changes in HDL influx rate; the vertical arrows represent changes with time due to intrinsic dynamics in the model tissue. (b) Plaques *A*–*E* plotted as a function of scaled time. As shown in the bifurcation diagram plaques *B*, *C* and *E* tend to the fixed equilibrium which has low foam cell density, but the density of foam cells in plaques *A* and *D* continue to grow. The scales on the axes are a qualitative indication only as there is limited information about the exact values of the input parameters in the model.

Fig 3(a) shows a bifurcation diagram, computed from the model. This diagram gives qualitative information about foam cell accumulation at different rates of HDL influx. The vertical black arrows represent model plaques (labeled *A* to *E*) that are developing over time each with a fixed rate of HDL influx. The direction of the arrows indicates whether the plaque is growing or shrinking. The horizontal dashed lines represent instantaneous changes in HDL influx. The solid curve represents the fixed equilibrium and is known mathematically, as an attractor. Plaques grow or shrink so as to get closer to the attractor. The dashed curve is known as the repellor. Plaques grow or shrink so that they move away from this curve. The dashed and solid curves meet at a point known as a bifurcation point or tipping point. We have labeled the rate of HDL influx at the bifurcation point Σ_*h*_. When the HDL influx rate is below Σ_*h*_, the density of foam cells in the model plaque will always increase as no equilibrium state exists. Fig 3(b) shows the accumulation of foam cells with time in each of the model plaques represented in Fig 3(a).

Plaque *A* represents a plaque with low HDL influx. Over time, the density of foam cells in Plaque *A* continues to grow, as shown in Fig 3(b). Plaque *B* on the other hand represents a plaque with a high rate of HDL influx. The density of foam cells in this plaque grows with time, but approaches the fixed equilibrium represented by the solid curve in Fig 3(a).

The effect of changing the rate of HDL influx in the model depends on the timing and magnitude of the change. Plaque *C* grows as the same rate as Plaque *A* initially, but after Plaque *C* has grown for a short time, the rate of influx of HDL into the plaque is instantaneously increased to the same HDL influx rate as Plaque *B*. When this happens, Plaque *C* loses foam cells via RCT and regresses to the fixed equilibrium foam cell density. Plaques *D* and *E* also grow in the same way as Plaques *A* and *C* initially, but the rate of HDL influx is increased later than for Plaque *C*. For Plaque *D*, the HDL influx rate is increased to the same value as Plaque *C* but the plaque continues to grow, albeit at a slower rate than for Plaque *A*. The difference between plaques *D* and *C* is the timing of the increase in HDL influx; in Plaque *C* the plaque was smaller when HDL influx increased and the new influx rate moves the plaque to between the dashed and solid curves in Fig 3(a). The plaque develops so that is gets closer to the fixed equilibrium, represented by the solid curve and further from the repellor which is represented by the dashed curve. Plaque *D* has grown sufficiently that, when HDL influx is increased, the plaque is above the dashed curve and so foam cells continue to accumulate as the plaque moves away from the repellor. If the rate of influx of HDL is increased at the same time as in Plaque *D* but by a larger amount, then the plaque will fall in the region between the solid and dashed curves and will regress (Plaque *E*).

In general, when the rate of HDL influx into the plaque is less than Σ_*h*_ (the HDL influx rate at the bifurcation point), there is no equilibrium and so the density of foam cells in the plaque continues to grow. If the rate of HDL influx is greater than Σ_*h*_ then there is an equilibrium or attractor where the density of foam cells in the plaque does not change. If the HDL influx remains constant and high enough, then a newly initiated plaque will approach this equilibrium and remain small. If HDL influx later drops below Σ_*h*_ the plaque will start to grow again. A subsequent increase in HDL influx may or may not lead to plaque regression and a consequent return to the small plaque with a fixed density of foam cells; this will depend on the timing and magnitude of the change in HDL influx.

**Fig 4:**
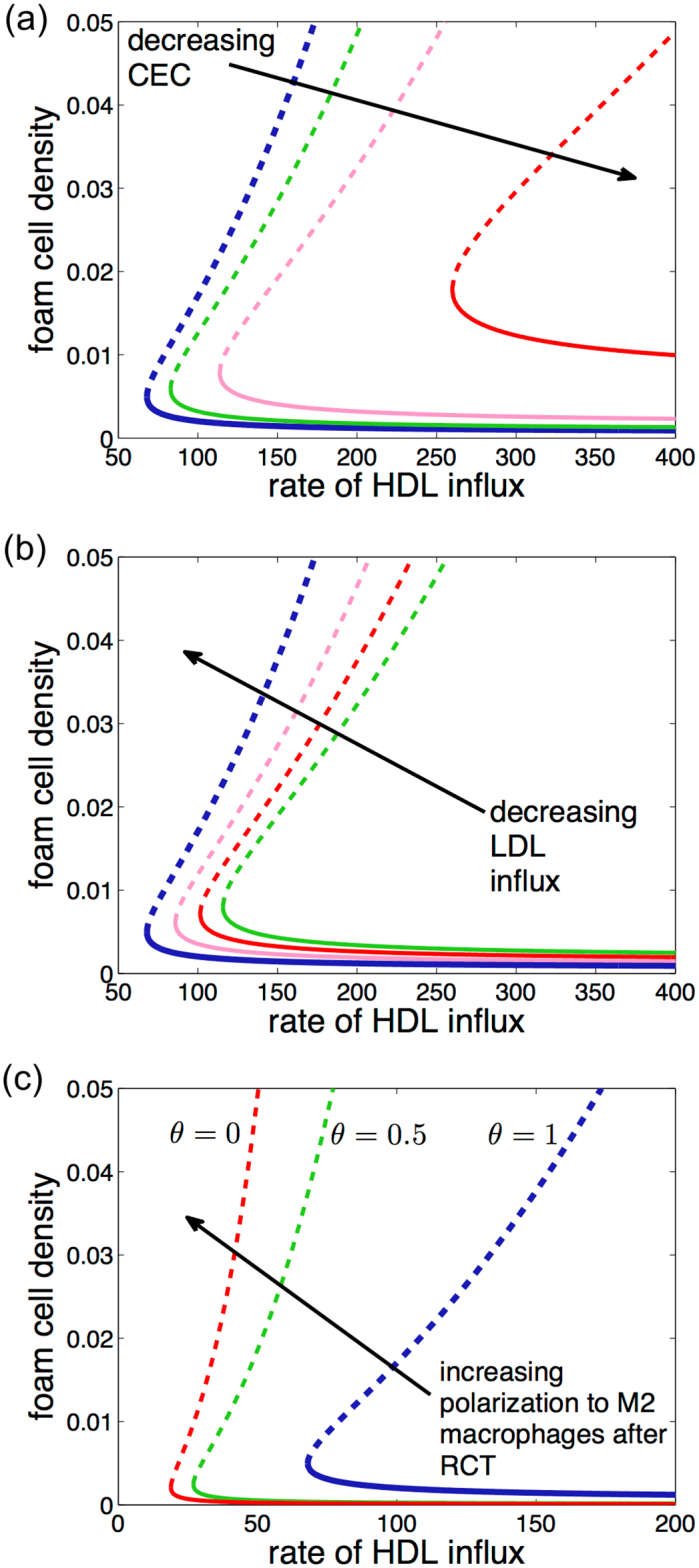
Equilibrium foam cell density for changing physiological parameters. Foam cell density at equilibrium (the attractor) and the repellor are plotted against HDL influx showing the effect of (a) increasing the cholesterol efflux capacity (CEC) of HDL particles; (b) increasing the influx of modLDL into the plaque and (c) increasing the proportion of macrophages that revert to M2 type after RCT. The attracting equilibria are indicated by the solid curves; the repellors by dashed curves. In (a), as cholesterol efflux capacity increases, the bifurcation point moves left so that the equilibrium exists for lower rates of HDL influx. In (b), as modLDL influx increases, the bifurcation point moves right so equilibrium only exists for higher rates of HDL influx. In (c), as the proportion of foam cells revert to M2 rather than M1 macrophages increases, the slope of the repellor curve increases so that it is more likely that a plaque will fall below the repellor curve if HDL influx is increased and consequently will regress. The blue curve in each plot is the same curve as in Fig 3(a).

**Fig 5:**
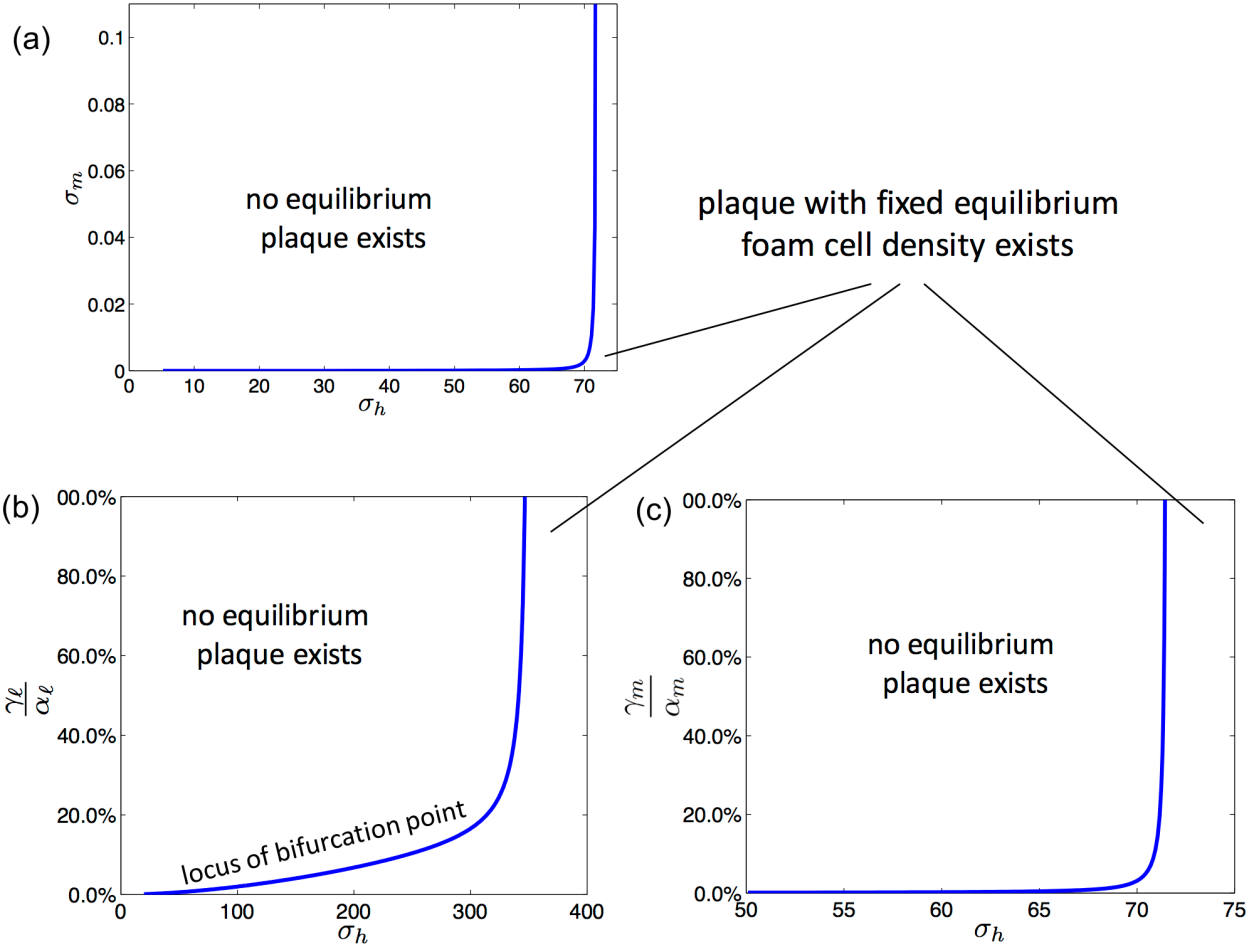
The effect of different HDL actions on equilibrium foam cell density. Plots showing where equilibrium plaques with a fixed density of foam cells exist as a function of *σ*_*h*_ the rate of influx of HDL and (a) *σ*_*m*_ which governs the rate of influx of monocytes and may be thought of as the availability of monocytes in the blood stream; (b) $\frac{\gamma_{\ell }}{\alpha_{\ell }}$ which governs the rate that LDL is modified and hence the rate that modLDL enters the model plaque; and (c) $\frac{\gamma_{m}}{\alpha_{m}}$ which governs the excitability of the endothelium in response to modLDL and its propensity to express adhesion molecules and thereby recruit macrophages to enter the lesion. In each plot, the curve is the locus of the bifurcation point.

The placement of the bifurcation point depends on various physiological parameters in the model (Fig 4). As the cholesterol efflux capacity of HDL particles increases, the bifurcation point moves left and so plaques can settle to a fixed equilibrium at lower rates of HDL influx. As the rate of LDL influx and consequent generation of modLDL increases, the bifurcation point moves right, so that the HDL influx rate that is required for a plaque to be at equilibrium increases. As the proportion of foam cells that revert to M2 type macrophages after reverse cholesterol transport increases, the repellor curve becomes steeper and so an increase in HDL influx is more likely to promote regression. Other parameters that affect the shape and placement of the curve in the bifurcation diagram, include the response of the endothelium to modLDL and the number of monocytes available in the blood stream (Fig 5).

**Fig 6:**
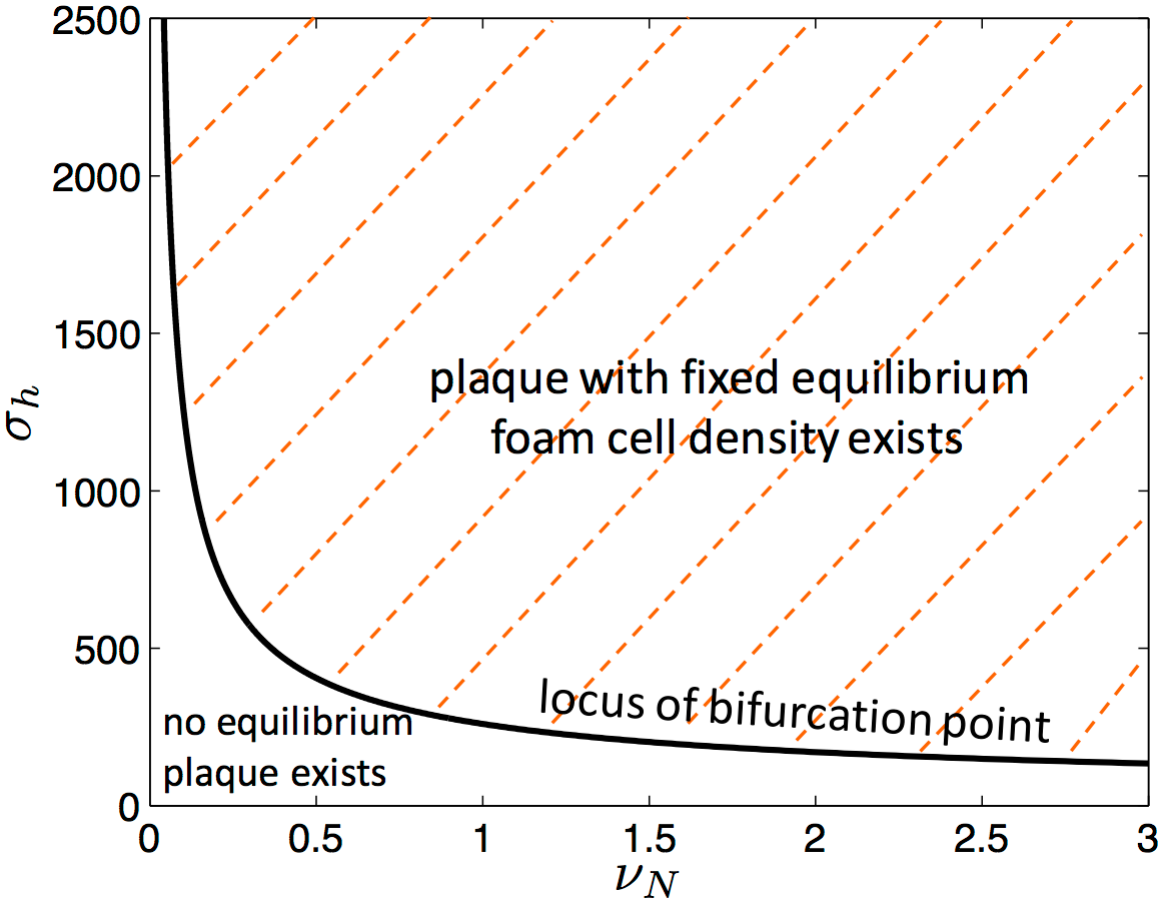
The existence of equilibrium plaque as a function of cholesterol efflux capacity and HDL influx. This plot shows where equilibrium plaques with fixed density of foam cells exist as a function of *σ*_*h*_ the rate of influx of HDL and *ν*_*N*_ which specifies the cholesterol efflux capacity of HDL and governs the rate of reverse cholesterol transport from foam cells. The solid curve is the locus of the bifurcation point; that is, of Σ_*h*_ as *ν*_*N*_ changes. This curve separates the region where an equilibrium plaque exists from the region where there is no equilibrium. As the cholesterol efflux capacity of the HDL particles decreases, the bifurcation point occurs at increasingly greater values of *σ*_*h*_ than when cholesterol efflux capacity is high. If cholesterol transport rates are very low then very high rates of HDL influx are required for a plaque to exist in equilibrium. On the locus of the bifurcation point, as *ν*_*N*_ → 0 then *σ*_*h*_ → ∞ which suggests that there it is impossible to have an equilibrium plaque when there is no reverse cholesterol transport.

Reverse cholesterol transport by HDL in the model is key to determining whether plaques continually grow or settle to an equilibrium where foam cells do not accumulate. Removing RCT from the model removes the attracting equilibrium so that plaques with fixed foam cell density cannot form and the only possibility is that plaques continue to grow. The effect of reducing CEC is shown in Fig 4(a). As CEC decreases, the bifurcation point moves right. When the HDL has no capacity to remove cholesterol from foam cells—that is, there is no RCT—then the bifurcation point and the fixed equilibrium disappear entirely. Similar information is presented in a different way in Fig 6. The anti-inflammatory effect of HDL on the endothelium and its anti-oxidative action alone is insufficient for plaque regression or, indeed, even for equilibrium in this model.

## Discussion

The results of this model suggest that the history of a plaque’s growth is as important as the rate of influx of HDL particles that the plaque experiences. If a plaque is well-established with a substantial foam cell density, then the model predicts that raising HDL availability by a moderate amount may not lead to regression but only to a slower rate of growth. Conversely, raising HDL influx very early in plaque growth or by a large amount will lead to regression.

The model results are consistent with experimental studies on rabbits and on ApoE^-/-^ and Ldlr^-/-^ mice. A study on rabbits that were fed an atherogenic diet for eight weeks and then injected weekly with HDL particles showed that the extent of fatty streaks in the aorta was about 50% lower than in the treatment group compared to both the baseline and the control groups after 30 days [13]. Another study using Ldlr^-/-^ mice injected with a human ApoA-I adenovirus after five weeks on an atherogenic diet before sacrifice four weeks later, showed a reduction in aortic lesion size of 70% compared to baseline groups and an even greater reduction compared to control groups [14]. A similar study on Ldlr^-/-^ mice with a short initiation phase did not produce regression but did demonstrate a greatly reduced progression rate so that 24 weeks after ApoA-I adenovirus injection the treatment group had lesions that were 50% smaller than the control group [15].

Studies with Ldlr^-/-^ or ApoE^-/-^ mice where HDL levels were increased after a period of plaque initiation of 6 months or longer followed either by human ApoA-I gene transfer [16, 17] or by transplant of the aorta into a wild-type mouse [18], did not produce regression in lesion areas even after several months of increased HDL levels but did show that plaques in the treatment group did not progress as rapidly as plaques in control animals. In another study [19] ApoE^-/-^ mice were given ApoA-I via infusion or gene transfer after either 8 or 34 weeks on atherogenic diets. Plaques in mice that were given infusions or gene transfer after 8 weeks grew more slowly than in untreated mice. There was no difference in plaque size between untreated mice and mice that were treated at 34.

The results of our model are consistent with the results of these studies which, together, suggest that an increase in HDL availability early in the life of a plaque is more likely to produce regression than a later increase in HDL influx.

**Fig 7:**
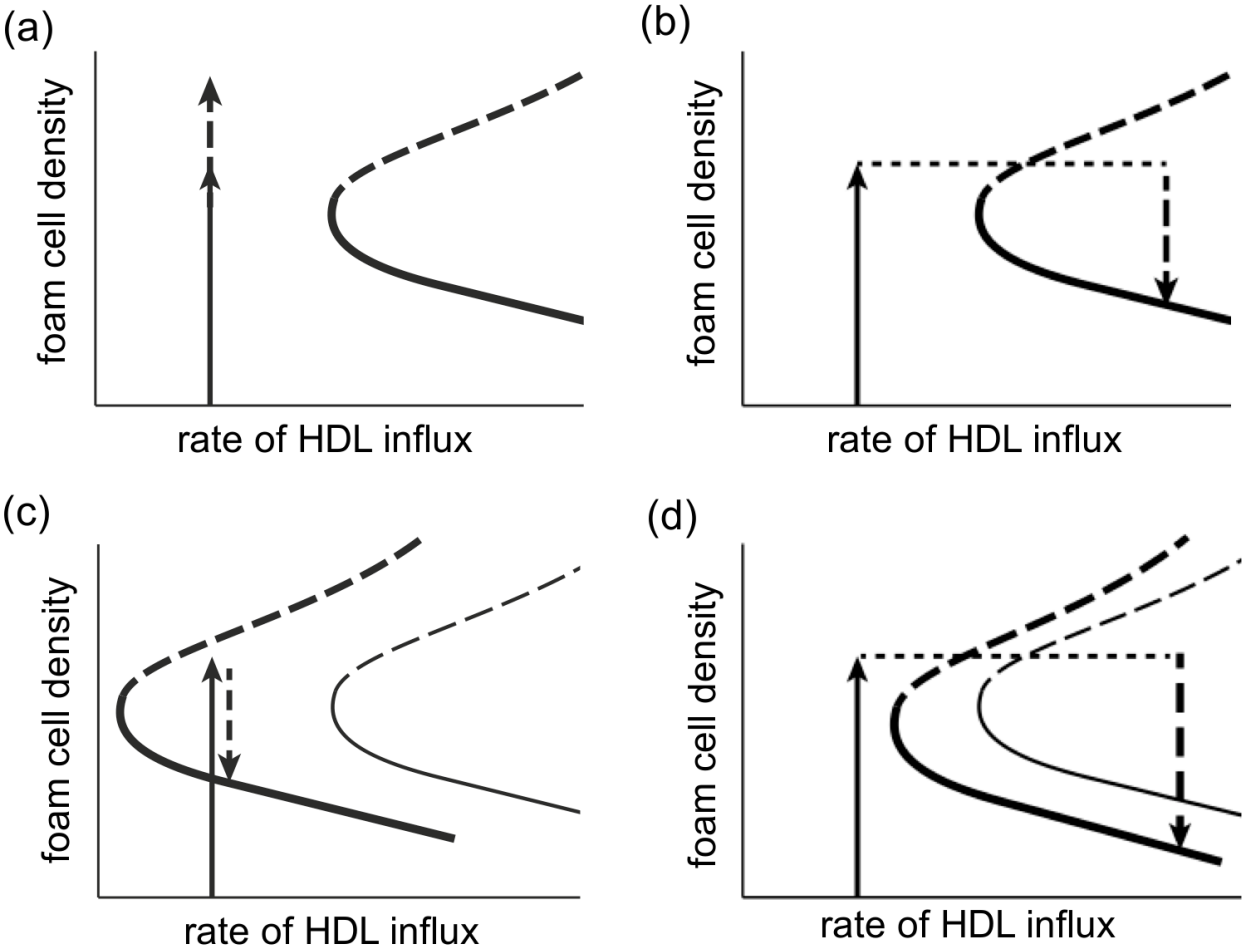
Diagrammatic representation of model predictions described in Table 1. Sketch of changes predicted by the model in plaque size after transplant into recipient mice: (a) ApoE-/-; (b) hAI/ApoE-/-; (c) ApoA-I-/-; (d) Wild Type. The curves in each plot represent the attractors and repellors. The solid curves represent the attractors or fixed equilibrium solutions and the dashed curves represent the repellors. Where there are two sets of curves, the heavier set of curves corresponds to the plaque after transplant and the lighter curve corresponds to the plaque before transplant. The solid arrow represents plaque growth before transplant and the dashed arrow represents post-transplant changes.

Reducing the influx of modLDL into the plaque moves the bifurcation point to the left so that the equilibrium with low foam cell density will exist for lower rates of influx of HDL (Fig 4(b)). This allows us to compare the predictions of the model to the results of a study by Feig et al. [29]. In this study ApoE^-/-^ mice were fed an atherogenic diet for 16 weeks and their aortas were each transplanted into genetically different mice so that the transplanted aortas were either in a similar environment to the donor mouse (the control) or in an environment with raised HDL-C compared to the donor mouse, lowered LDL-C or both. (We assume here that these correlate well with HDL and LDL particle number [9].) The recipient animals were on a chow diet and sacrificed after one week. Table 2, together with Fig 7, summarizes the results of this study and their interpretation according to this model. In each case, the experimental results can be explained qualitatively using the model and these explanations are consistent with one another.

**Table 2:** Model results compared with experiments. This table shows the results of different treatments by Feig et al [29] and the corresponding prediction of the model.

Recipient mouse	Change in blood HDL-C and LDL-C.	Observed change in lesion area from baseline size.	Figure with bifurcation diagram.	Explanation from model.
ApoE^−/−^	No change: HDL-C remains low; LDL-C remains high	Increasing trend	Fig 7(a)	No change in plaque behaviour. Plaque continues to grow.
hAI/ApoE^−/−^	HDL-C rises to normal levels; LDL-C remains high	Significant decrease (*P* < 0.05)	Fig 7(b)	Change in HDL levels puts plaque to the right of the bifurcation point and below repellor so the model plaque regresses.
ApoA-I^−/−^	HDL-C remains low; LDL-C falls to low levels	Decreasing trend	Fig 7(c)	Decrease in LDL moves the curve left; the plaque can now regress to the equilibrium.
Wild Type	HDL-C rises to normal levels LDL-C falls to normal levels	Significant decrease (*P* < 0.05)	Fig 7(d)	Decrease in LDL moves curve left. Increase in HDL moves the plaque right. Plaque tends to an equilibrium that is smaller than for the ApoAI^−/−^ recipient

In contrast to much of the research with animal models, clinical studies on HDL-raising in humans are usually conducted in patients with late stage plaque, usually following the onset of clinical symptoms such as an acute coronary event of other evidence of vascular disease. The modelling suggests that the large reduction seen in plaques in animal models are unlikely to be observed in these late-stage plaque studies. The complicated structures of late-stage plaque may also be a confounding factor. Generally speaking reductions in volumes in advanced human plaques are small compared to the reductions in the volume of early plaques seen in animal studies. Typically patients’ HDL particle numbers are raised by weekly infusions for up to two months of an ApoA-I mimetic [41], an ApoA-I/phospolipid complex [42] or of their own delipidated HDL [43] and the plaque volume in a target coronary artery was measured at the end of the treatment. These treatments, at best produce a reduction of less than 7% in plaque volume relative to baseline. The only exception to this is the study by Shaw et al [44] which showed a reduction of 62% in macrophage lipid content and 58% in macrophage size relative to the placebo group in plaques that were excised from the femoral artery a week after infusion with reconstituted HDL.

The existence of the bifurcation point at the critical value of HDL influx Σ_*h*_ suggests why low HDL-C in humans (which is assumed to correlate with HDL particle number or action [9]) is consistently correlated with increased risk of CVD, but high HDL-C is not necessarily correlated with low CVD risk [12]. If this switch between continual plaque growth with no equilibrium plaque size and an accessible equilibrium exists *in vivo*, then further increases in HDL particle numbers which usually correlates with increases in HDL-C [9], provide very little benefit once HDL influx is sufficiently high for there to be enough HDL for a plaque to settle at equilibrium.

The model also highlights the key role of the cholesterol efflux capacity (CEC) of HDL. If CEC is high then plaques will reach an equilibrium for lower values of HDL influx than if CEC is low (Fig 6). When CEC is low, a plaque is more likely to continue to grow. This result agrees with population-level data on the relation between CEC and heart attack and stroke [45].

There are only a few previous modelling studies which include HDL [23, 46]. There is evidence in these studies that bifurcations, similar to those found here, exist in their models and can determine the fate of a plaque when HDL input or efficacy is changed. Cohen *et al*. [23] present bifurcation diagrams which show that bifurcation points exist and that these can lead to sudden switches between plaques being at equilibrium and growing unboundedly. In Cohen *et al*.’s model, high rates of macrophage influx lead to continual growth but low rates of macrophage influx lead to plaques settling to one of two possible equilibriums. However, these bifurcation diagrams depend on the rate of macrophage influx rather than the rate of HDL influx. Because in Cohen *et al*.’s model macrophage influx is implicitly dependent on HDL concentration, this gives a limited insight into the anti-inflammatory effect of HDL on the endothelium in promoting plaque equilibrium. Cohen *et al*. [23] further show that the effects of changing the rate of macrophage influx depends on when the change is made. If the rate of macrophage influx is reduced early in the plaque’s life, a rapidly growing plaque will settle to equilibrium, but if the rate of macrophage influx is reduced later in the plaque’s life in their model, then the plaque continues to grow, but at a slower rate.

Friedman and Hao [46] show that small increases in HDL efficacy in their model lead to a reduction in the rate of plaque growth although plaques do continue to grow, but large increases in HDL efficacy lead to a reduction in plaque weight and macrophage density. It is not clear whether these regressing plaques reach an equilibrium in their model.

Both these models and the model that we present in this paper suggest that nonlinearities in the dynamics of HDL action in plaques may lead to sudden changes in plaque behaviour after periods of consistent growth or equilibrium when the plaque environment changes due, for example, to the change in HDL efficacy with age [47] or decrease in LDL influx due to successful treatment with statins.

There is currently ongoing interest in the infusion of HDL particles or HDL mimetics to promote plaque regression [8, 48, 49]. This study suggests that some patterns of dosage will be more successful than others. It also suggests that it may be possible to engineer a change in the fate of a plaque from continual growth to regression to a small plaque in equilibrium.

**Fig 8:**
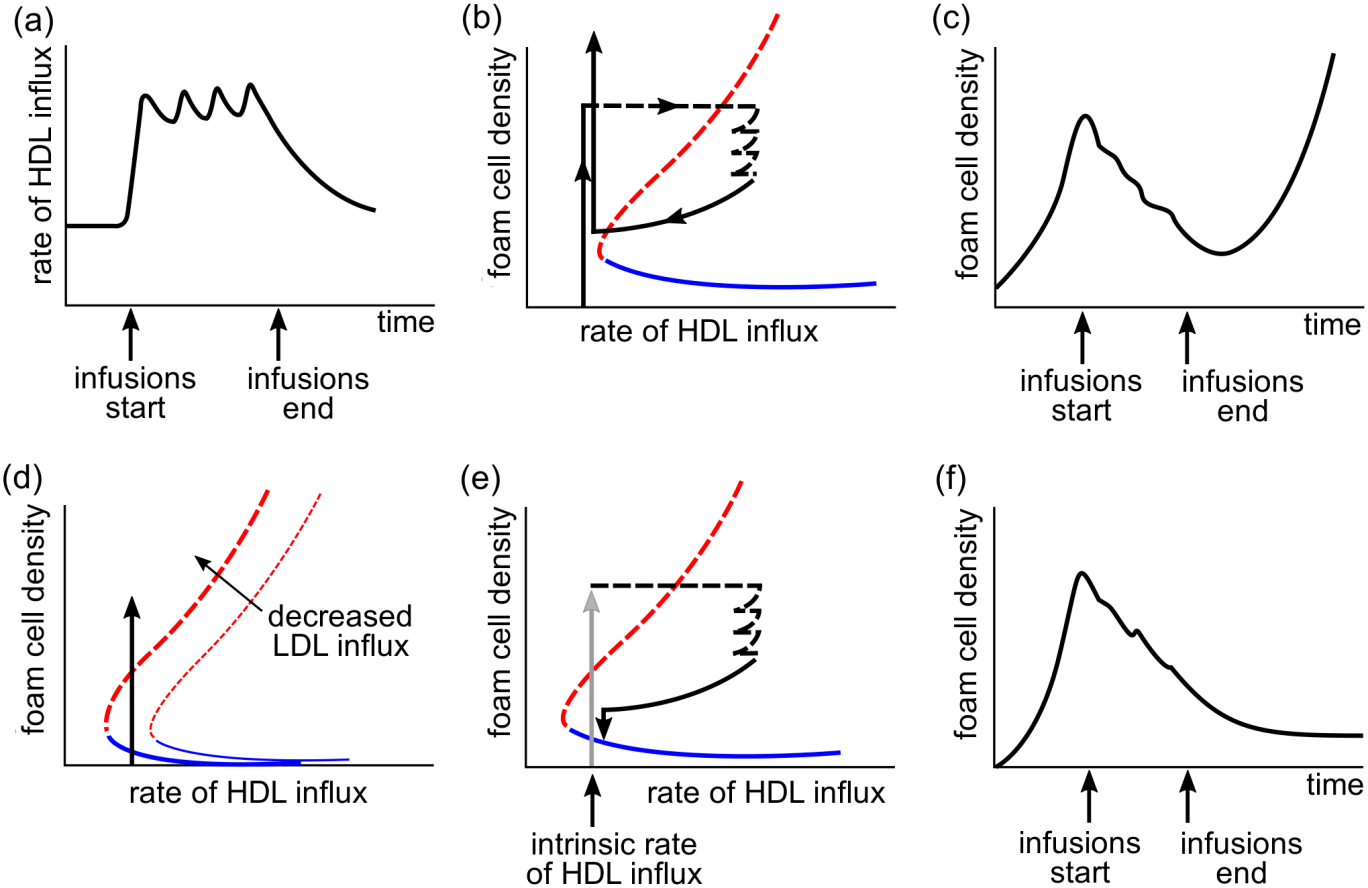
Diagram illustrating of the effect of treatment by raising HDL influx into plaques, showing schematically the effect of a period of infusion of HDL that leads to increased HDL influx into the plaque, both with and without a simultaneous lessening of LDL influx. (a) HDL influx into the plaque as a function of time where there is a period of regular infusions, preceded and followed by a period of no infusions. (b) Sketch of the bifurcation diagram showing the solution for the plaque under the HDL influx regime illustrated in (a) when LDL influx doesn’t change. The solution evolves over time along the black curve in the direction of the arrows. When the infusions are completed the plaque returns to a state where it cannot reach the attractor but continues to grow; (c) Sketch of plaque size as indicated by foam cell density as a function of time under the HDL influx regime illustrated in (a) when LDL influx doesn’t change. This plot corresponds to the progress of the plaque shown in (b) but here foam cell density is plotted explicitly as a function of time; (d) sketch of the changes in the bifurcation diagram when LDL influx is decreased before HDL infusion begins. (This might model the introduction of statin therapy for example.) The black arrow represents plaque growth before LDL influx decreases. We assume that the LDL influx rate is changed after the plaque has developed sufficiently so that the solution lies above the repellor on the new bifurcation curve. (e) Sketch of the bifurcation diagram showing the solution for the plaque under the HDL influx regime illustrated in (a) when the LDL influx is changed before HDL infusion is started. The grey arrow represents plaque growth prior to LDL increase and HDL infusion. Since the untreated level of HDL influx now lies to the right of the bifurcation point, the plaque will settle to an equilibrium once infusions stop since HDL infusion has reduced the size of the plaque so that the solution can approach the attractor and reduced LDL levels ensures that the attractor is now accessible when there is no HDL infusion. This new equilibrium is stable as long as LDL influx into the plaque remains low.; (f) Sketch of plaque size as indicated by foam cell density as a function of time under under reduced LDL influx and the HDL influx regime illustrated in (a). This plot corresponds to the progress of the plaque shown in (e) but here foam cell density is plotted explicitly as a function of time.

Fig 8 shows the potential effect of treatment on a model plaque. For most treatments to be effective, the model suggests that the increase in HDL levels must be maintained; that is, treatment will usually need to be ongoing, in the same way that statin treatment to reduce levels of LDL cholesterol in the blood is long term. For regression to occur in the model, the increase in sustained HDL influx rates into the plaque must be sufficiently large that the solution for the model plaque lies between the repelling and attracting solutions in the bifurcation diagram. If the increase in influx of HDL is sufficiently large to drive the plaque below the threshold imposed by the repelling solution, then the plaque will regress (Fig 8(b) and 8(c)). This is similar to model plaques *C* and *E* in Fig 3.

If the HDL influx increase is not large enough, then the only change that might be observed is a decrease in growth rate. This is similar to model plaque *D* in Fig 3. A failure to create sustained levels of HDL activity in plaques may be behind null results obtained in studies on humans where HDL was infused weekly [41]. Other studies suggest that apo-AI (the protein in HDL) decreases exponentially from the time of infusion with a half-life of at most 36 hours [50] so that HDL concentration in the bloodstream returns to baseline levels after about 3 days and so a weekly infusion which may result in significant variations in HDL availability may constantly switch the plaque between decreasing to an attracting equilibrium and increasing without bound over the weekly treatment cycle.

The model does, however, suggest that it is possible that increasing HDL influx into the plaque by increasing HDL via infusion for a limited time, may sometimes lead to permanent plaque regression. Consider a model plaque that is experiencing growth at a level of HDL influx where there is an equilibrium solution (Fig 8(e) and 8(f)). This is illustrated in Fig 8(d). This plaque may, for example, have started growing when the modLDL influx rate was higher and so the bifurcation curve has moved to the left since the plaque was initiated. This might model the commencement of statin treatment or the effect of lifestyle changes, for example. If the plaque is given a sufficiently high sustained HDL influx through HDL infusion, the model plaque will move to the region between the repellor and the equilibrium solution and regress towards the equilibrium solution. If HDL infusion ceases and the HDL influx returns to its original state, then the plaque will remain at equilibrium and remain small. This effect requires an ongoing low modLDL influx into the plaque and a temporary increase in HDL that is sufficiently high and lasts long enough to “reset” the plaque to the regressed equilibrium state.

Large clinical trails have commenced for infusion of HDL mimetics have the application of gene therapy as treatment for late stage plaques [48, 49, 51]. Preliminary results suggest that regular, ongoing infusions of a HDL mimetic can reduce the area of a plaque by about 6.7% on average [52]. However, most of these trials are being conducted on patients with advanced plaques which have necrotic cores. Our model predicts that a sufficiently large increase in the action of HDL in plaques, whether due to increasing the concentration of HDL particles or increasing their efficacy will reduce the lipid burden of plaque macrophages and foam cells. However, to reduce the size of advanced plaques, extracellular lipid needs first to be ingested by cells before it can undergo reverse cholesterol transport by HDL. This ingestion of extracellular lipid may be a comparatively slow process [53] and so, although the nonlinear dynamics of HDL action that we explore in this study may be a factor in the low degree of regression of advanced plaques, the presence of the necrotic core, which is not included in this model, may be another fundamental cause of low rates of regression in advanced plaques which contain extracellular lipid.

The computational model in this study has many nonlinear terms that model different facets of cell and cytokine action in plaque growth and further work is required to determine which of these dominate in plaque formation and regression. Accurate values for most of the parameters in the model cannot yet be obtained from the experimental literature and consequently many of these are estimates or values that are obtained from other contexts or from general considerations of how cells and cytokines behave. Nevertheless, this analysis strongly suggests that the effects of nonlinear interactions may be significant in the dynamics of plaque formation and regression. Several laboratory research groups [54–56] have recently begun to characterize macrophage movement and plaque growth in terms of dynamics. It is important to understand, in such a complicated and multifactorial process, that nonlinear interactions occur and may lead to discontinuous switches and changes due to bifurcations. These bifurcations will not occur only for changing levels of HDL influx but may also occur for other physiological variables, such as the availability of monocytes in the blood stream or the influx of LDL particles into the plaque [57, 58].

As yet, there is only indirect evidence that these types of nonlinear switches occur in plaques *in vivo*, but if they do, there is likely to be implications for the management of plaque growth and for strategies to promote plaque regression. This study, for example, suggests, if the modelling is valid, that the influx rate of HDL into the arterial wall must either be increased sufficiently early in the life of the plaque or be sufficiently substantial for the plaque to regress, that is, for the attracting equilibrium to be accessible under the newly increased rate of HDL influx. This raises questions about the dynamics of HDL action and RCT in plaques in addition to the plethora of questions that already surround drug interventions to increase HDL levels in patients at high risk of CVD.

In conclusion this study suggests, via a computational model that there may be a bifurcation point in plaque growth. (The bifurcation point is also called the tipping point in other scientific contexts.) Plaques with HDL influx below this point will continue to grow but plaques with HDL influx above this point may settle to an equilibrium where they do not grow. The model also suggests that both the timing and magnitude of any increase in HDL influx into plaque will determine whether or not a plaque regresses or continues to grow. Whether or not a plaque remains small or regresses to equilibrium depends also on the influx of LDL into the plaque and the cholesterol efflux capacity of the functional HDL. These results suggest that therapy that raises HDL may be most effective in preventing the growth of small plaques rather than promoting the regression of large plaques.

A theory of the dynamics of early plaque growth and regression has the capacity, not only to generate new hypotheses, but also to provide explanations of anomalous observations and a framework to synthesize research results. Developing this theory and devising biologically valid computational and mathematical models to encapsulate theoretical ideas will require care and persistence and is unlikely to be simple or straightforward. This analysis is one early step in the journey.
